# Genome-Wide Control of the Distribution of Meiotic Recombination

**DOI:** 10.1371/journal.pbio.1000035

**Published:** 2009-02-17

**Authors:** Corinne Grey, Frédéric Baudat, Bernard de Massy

**Affiliations:** Institut de Génétique Humaine, UPR1142-CNRS, Montpellier, France; National Cancer Institute, United States of America

## Abstract

Meiotic recombination events are not randomly distributed in the genome but occur in specific regions called recombination hotspots. Hotspots are predicted to be preferred sites for the initiation of meiotic recombination and their positions and activities are regulated by yet-unknown controls. The activity of the *Psmb9* hotspot on mouse Chromosome 17 (Chr 17) varies according to genetic background. It is active in strains carrying a recombinant Chr 17 where the proximal third is derived from *Mus musculus molossinus*. We have identified the genetic locus required for *Psmb9* activity, named *Dsbc1* for Double-strand break control 1, and mapped this locus within a 6.7-Mb region on Chr 17. Based on cytological analysis of meiotic DNA double-strand breaks (DSB) and crossovers (COs), we show that *Dsbc1* influences DSB and CO, not only at *Psmb9*, but in several other regions of Chr 17. We further show that CO distribution is also influenced by *Dsbc1* on Chrs 15 and 18. Finally, we provide direct molecular evidence for the regulation in *trans* mediated by *Dsbc1*, by showing that it controls the CO activity at the *Hlx1* hotspot on Chr 1. We thus propose that *Dsbc1* encodes for a *trans*-acting factor involved in the specification of initiation sites of meiotic recombination genome wide in mice.

## Author Summary

In many organisms, an essential feature of meiosis is genetic recombination, which creates diversity in the gametes by mixing the genetic information from each parent into new combinations. Reciprocal recombination, or crossovers, also play a mechanical role during meiosis and are required for the proper segregation of homologous chromosomes to the daughter cells. Crossovers do not occur randomly in the genome but rather are clustered in small regions called hotspots. The factors that determine hotspot locations are poorly understood. We have analyzed a particular recombination hotspot in the mouse genome, called *Psmb9*, and showed that its activity is induced by a specific allele of a locus that we have mapped and named *Dsbc1*, for Double-strand break control 1. We have analyzed the properties of *Dsbc1* both by the direct detection of recombinant DNA molecules in specific regions and by chromosome-wide cytological detection of proteins involved in recombination. Our results show that *Dsbc1* acts genome wide and regulates the distribution of crossovers in several regions on different chromosomes, at least in part by regulating the initiation step of meiotic recombination characterized by the formation of DNA double-strand breaks.

## Abstract 3

*Dsbc1* is a novel locus involved in controlling the localization of meiotic recombination events in the mouse genome.

## Introduction

During the first meiotic prophase, chromosomes undergo a series of unique events that lead to the formation of stable connections between homologous chromosomes (homologs), which are required for the reductional segregation at the first meiotic division. In most species, these connections result from the formation of at least one reciprocal recombination event or crossover (CO) per chromosome arm between chromatids from homologs, stabilized by the maintenance of cohesion between sister chromatids [1]. In addition to this mechanical role, COs increase genetic diversity by reshuffling alleles in the genome and thus are thought to increase the efficiency of selection [2].

The main lines of the mechanism of CO formation have been uncovered in Saccharomyces cerevisiae and Schizosaccharomyces pombe and are conserved in other eukaryotes [3]. Meiotic recombination is induced by the formation of localized DNA double-strand breaks (DSBs), catalyzed by the evolutionary conserved Spo11 protein at the leptotene stage, corresponding to the beginning of the meiotic prophase [4]. These DSBs are repaired by homologous recombination preferentially with a chromatid from the homologous chromosome. This homologous repair leads to gene conversion with CO or to gene conversion without CO (noncrossover or NCO). One important implication of this mechanism is that the number and distribution of COs depend both on the number and distribution of DSBs and on the proportion of DSBs repaired towards COs or NCOs [5,6].

Direct analysis of DSB events in yeasts has shown that initiation occurs nonrandomly in the genome and preferentially in small regions known as recombination hotspots. In S. cerevisiae, DSBs occur almost exclusively in intergenic regions adjacent to transcription promoters [7,8]. When tested, the transcription activity of the adjacent promoter was found not to be required, although binding of transcription factors can stimulate DSB formation [9]. At several hotspots, DSBs were found to occur in accessible regions of the chromatin [10,11]. In addition to these local constraints, another control appears to act at the level of chromosomal domains. In fact, an active initiation site loses its activity when inserted in a region with no or low DSB activity, suggesting a role for higher order chromosome structure [12]. In S. pombe, the *ade6-M26* initiation site is located next to the binding site of the Atf1/Pcr1 transcription factor, and DSB formation is enhanced upon binding of Atf1/Pcr1, which induces a reorganization and modification of the chromatin [13–15]. Open chromatin configurations were also detected at several other DSB sites [16]. Recently, the global analysis of DSB formation in S. pombe has shown that DSBs are clustered in small intervals separated by large regions with low or no DSB activity. DSBs do not necessarily occur next to transcription promoters; they appear to preferentially occur in large intergenic regions [17]. Thus, in both yeasts, the parameters that define an initiation site are not defined by simple features of the primary DNA sequence; they are somehow related to a control of accessibility. This notion is indeed supported by experiments in S. cerevisiae that show that DSBs can be induced by targeting Spo11 to a Gal4 binding site through the expression of a Gal4-Spo11 fusion protein [18]. In such situations, DSBs occur adjacent to the Gal4 binding site. Interestingly, constraints imposed by chromosomal domains are still observed in this case since Gal4-Spo11-induced DSBs do not form in domains with low natural DSB activity [19].

In other organisms, DSBs have not been detected directly and the distribution of recombination is derived from mapping COs, cytologically, genetically or by population diversity analysis [20,21]. In humans, CO distribution, which has been analyzed throughout the genome at high resolution, is not random and shows a specific clustering in small intervals (up to 1–2 kb long) separated by larger regions (50–100 kb) with no or low CO activity [22,23]. Interestingly, a common sequence motif has been identified in a substantial fraction of human recombination hotspots [24]. Moreover, when measured over several Mb-long domains, CO activity varies, some domains showing high (jungle) or low (desert) CO rates [25,26]. In mice, low-resolution analysis of CO also suggests similar large-scale constraints [25,27]. Several hotspots have been localized and shown to have similar properties to those described in the human genome, in particular with a tight clustering of COs in 1–2-kb intervals [21,28,29]. A recent high-resolution CO map of mouse Chromosome 1 (Chr 1) has shown that this pattern of distribution of recombination is indeed a general feature in the genome and has led to the identification of several new hotspots in the mouse genome [30].

Interestingly, variations of CO activities between individuals, sexes, and populations in human, or strains and sexes in mice have provided additional clues to identify factors that contribute to hotspot localization and activity [31]. The difference in CO activity between sexes reveals a DNA sequence-independent control of CO sites for which the mechanism, potentially related to epigenetic modifications, remains to be understood. Variations in genetic maps in different mouse hybrids have been observed [32] and could be the consequence of heterozygosity and/or of genetic factors. The contribution of genetic determinants on CO distribution and hotspot activity has been recently deduced from the analysis of interindividual variations in humans [33]. Variation of hotspot activity according to genotypes has also been shown at several sites in the mouse genome [29,34–36].

In order to analyze the molecular basis of the control of hotspot activity, we have taken advantage of the unique properties of the *Psmb9* hotspot located on Chr 17 in the mouse genome. *Psmb9* hotspot activity is influenced by several factors such as sex and genotype [37]. We have shown that both *cis-* and *trans*-acting elements control the initiation activity of *Psmb9*. The regulation in *trans* was deduced from the observation that a specific haplotype on Chr 17 on one homolog could activate initiation at *Psmb9* on the other homolog [38]. In the present study, by generating new recombinant chromosomes, we have been able to locate the genetic element responsible for *Psmb9* activity within 6.7 Mb on Chr 17. We show that this element actually acts by regulating the distribution of COs genome wide. Specific analyses on Chr 17 suggest that the regulation acts at the level of initiation. These results uncover a novel genetic control of the distribution of meiotic recombination events, predicted to influence where initiation occurs without altering the total CO activity along chromosomes. A similar control depending on a locus mapped in an overlapping region is described in the accompanying paper (Parvanov et al. [39]).

## Results

### The *Psmb9* Hotspot Is Controlled by a Long Distance–Acting Element

The activity of the *Psmb9* hotspot has been shown to depend on the presence of the *wm7* haplotype derived from an isolate of *Mus musculus molossinus* [36,40]. Both *Psmb9* and the *wm7* haplotype are located on Chr 17 and the *wm7* fragment required for *Psmb9* activity extends from the centromere of Chr 17 to the center of the hotspot [37,38]. The *wm7* haplotype needs to be present in only one parental chromosome and leads to a 2,000-fold increase in CO activity at *Psmb9* in hybrids in which the homologous chromosome can be from different genetic origins [41]. The molecular analysis of CO and NCO events at *Psmb9* in hybrids with or without a chromosome carrying the *wm7* haplotype showed that the variation of CO frequencies were most likely due to variations in initiation frequencies, given the parallel variations of NCO events. Furthermore, these analyses revealed that the presence of the *wm7* haplotype on one homolog was sufficient to induce initiation at *Psmb9* on both homologs, indicative of a regulation acting in *trans* [38].

**Figure 1:**
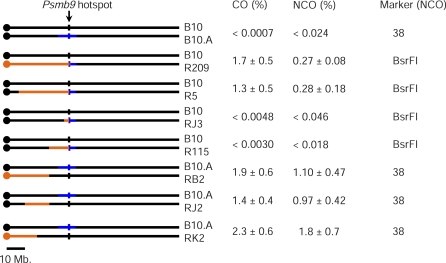
CO and NCO Events at *Psmb9* Are Activated by a Long Distance–Acting Element from the *wm7* Haplotype The origin of the Chr 17 fragments in the different recombined lines is represented: black, B10 (B10 or B6 for R115) (haplotype *b*); blue, A (haplotype *a*); and orange, SGR (haplotype *wm7*). The 95% confidence intervals for recombinant product frequencies are calculated as described in [38]. NCOs were detected either at the BsrFI site or the site “38,” depending on which is heterozygous in each hybrid. Values for B10xB10.A and B10xR209 are from [38].

To determine more precisely the position of the genetic element responsible for the high level of recombination at the *Psmb9* hotspot, we generated recombinant chromosomes carrying shorter *wm7* fragments by genetic crosses. The starting strain was B10.A(R209), named R209. In this strain, the *wm7* haplotype extends from the centromeric region of Chr 17 to *Psmb9*. R209 was crossed with C57BL/10 (B10), and progeny carrying recombined Chr 17 were screened. The extent of the *wm7* fragments in the progeny were determined by genotyping using microsatellite markers, and the derived strains carrying recombined Chr 17 were mated with either B10 or B10.A in order to generate hybrids in which sperm DNA could be assayed for COs and NCOs at *Psmb9*. Six new strains carrying different segments of the *wm7* haplotype (R5, RJ3, R115, RB2, RJ2, and RK2) were thus generated (Figure 1).

Unexpectedly, in the hybrids B10xR115 and B10xRJ3, neither CO nor NCO products could be detected at *Psmb9*, indicating that in these hybrids, initiation activity had been lost on both homologs and that the sequences of *wm7* surrounding the hotspot are not sufficient for inducing its activity. On the R115 chromosome, the *wm7* fragment extends 10.2–14.1 Mb to the left of *Psmb9*. In contrast, B10xR5 hybrid had high CO and NCO frequencies at *Psmb9* indicating that a remote element was required for *Psmb9* activity. Indeed, in the RB2xB10.A hybrid in which the RB2 chromosome contains a *wm7* fragment from the centromeric region up to 9.3–10.2 Mb from *Psmb9*, high frequencies of COs and NCOs, similar to the ones of B10xR209, were detected, demonstrating that an element from the *wm7* haplotype located more than 10.2 Mb away from the hotspot is necessary and sufficient for its recombinogenic activity. Two additional recombinant Chr 17, derived from RB2 (named RJ2 and RK2), and carrying shorter *wm7* fragments, retain full *Psmb9* recombinogenic activity. Therefore, the genetic element necessary and sufficient for the activation of the *Psmb9* recombination hotspot is located in a 6.7-Mb region of the *wm7* fragment between positions 10.1 and 16.8 Mb of Chr 17.

### The *Psmb9* Control Element Influences CO Activity in Several Other Regions on Chr 17

**Figure 2:**
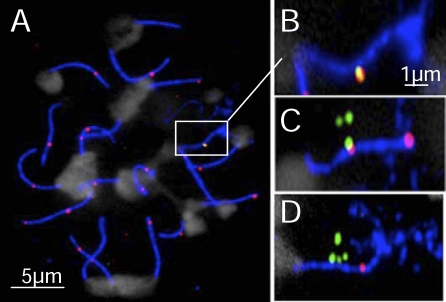
MLH1 Foci at *Psmb9* Correlate with CO Activity (A–D) Immuno-FISH assay on adult pachytene spermatocyte spreads. SYCP3 (blue), MLH1 (red), Chr 17 probe (mix of BACs RP23-10B20 and RP24-67L15; blue), *Psmb9* PCR fragments probe (green), and DAPI staining (white). (A) Whole nucleus. (B and C) Chr 17 from nuclei showing colocalization between MLH1 and *Psmb9*, with distances of 70 nm and 200 nm, respectively. (D) Chr 17 without MLH1/*Psmb9* colocalization, with four *Psmb9* foci corresponding to the four chromatids.

In order to analyze the mechanism involved in *Psmb9* activity, we designed an approach to determine whether the effect of the *wm7* haplotype was specific to *Psmb9* or if it could affect other regions on Chr 17. In order to address this question, we monitored CO activity by the detection of MLH1 foci on Chr 17 synaptonemal complex (SC) by immunofluorescent in situ hybridization (immuno-FISH) (Figure 2A). MLH1 foci are detected from middle to late pachytene, localize to future sites of chiasmata [42], and have the same global distribution as COs obtained from the genetic map [43]. Given the high CO frequency at *Psmb9*, which is 1.9 ± 0.6% in sperm DNA from the RB2xB10.A hybrid (Figure 1), one predicts that about 3.8 ± 1.2% of pachytene spermatocytes should have a MLH1 focus colocalizing with *Psmb9* in this hybrid. We thus measured the positions of MLH1 and *Psmb9* signals in B10xB10.A and RB2xB10.A hybrids. The signals were considered to colocalize when the centers of at least one *Psmb9* focus and of the MLH1 focus on Chr 17 were separated by less than 300 nm (Figure 2B–2D). RB2xB10.A hybrid had a higher MLH1/*Psmb9* colocalization frequency compared to B10xB10.A (4.7 ± 1.2% vs. 1.4 ± 1.2%; Fisher exact test, *p*-value = 0.021). This suggests that 3.3 ± 1.5% colocalization is due to the activity of *Psmb9*, which is in good agreement with the predicted value based on CO frequency. This analysis also indicates that the frequency of colocalization by chance is around 1.4%. The number of detectable *Psmb9* foci per nucleus varied, as expected from one to four, depending on the relative positions of the four chromatids. RB2xB10.A mice showed a lower proportion of nuclei with two, three, or four foci as compared to B10xB10.A mice (Figure S1), indicating that the higher MLH1/*Psmb9* colocalization frequency observed in the RB2xB10.A hybrid compared to B10xB10.A could not be explained by a higher average number of *Psmb9* foci per nucleus. Taken together, these results validate our immuno-FISH approach and provide further evidence that MLH1 foci are faithful markers of CO events at our level of resolution.

We then tested whether the increased activity at *Psmb9* could reflect a global increase of CO frequencies on Chr 17 by comparing the average number of MLH1 foci on Chr 17 in B10xB10.A and RB2xB10.A hybrids. These were found to be identical (Table 1). SC length was also identical in the two hybrids (Table 1). In order to identify potential differences in SC compaction, we determined the positions of three different bacterial artificial chromosome (BAC) probes along the SC of Chr 17. In both hybrids, all three BAC probes localized to the same positions (Figure S2, Table 1), indicating that chromosome compaction along the SC is not significantly different between B10xB10.A and RB2xB10.A hybrids.

**Table 1:**
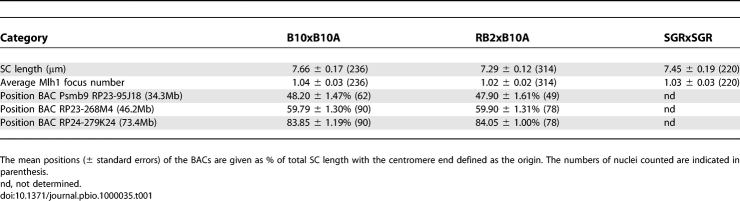
SC Length, MLH1 Foci, and BAC Positions on Chr 17 in Different Hybrids

**Figure 3:**
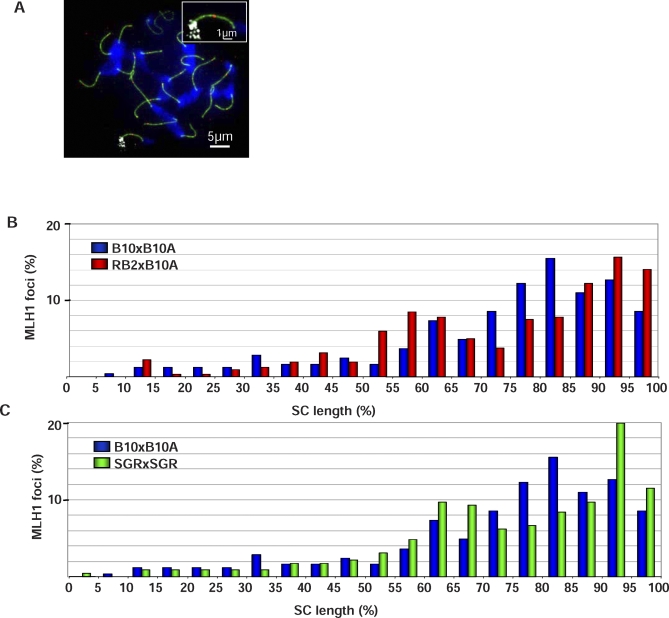
Different Distributions of MLH1 Foci on Chr 17 in B10xB10.A, RB2xB10.A, and SGRxSGR (A) Pachytene spermatocyte labeled with SYCP3 (green), MLH1 (red), and Chr 17 probe (mix of BACs RP23-10B20 and RP24-67L15; white) and stained with DAPI (blue). The region of Chr 17 is enlarged in the inset. (B) Percentage of MLH1 foci per 5% SC length intervals on Chr 17 in B10xB10.A and RB2xB10.A hybrids (*n* = 245 and 320 foci, respectively). (C) Percentage of MLH1 foci per 5% SC length intervals on Chr 17 in B10xB10.A and SGRxSGR hybrids (*n* = 245 and 226 foci, respectively).

In order to test whether the *Psmb9* control element could affect CO frequencies elsewhere on Chr 17, we mapped MLH1 foci along SC of Chr 17 in B10xB10.A and RB2xB10.A hybrids (Figure 3A). Surprisingly, the global distribution of MLH1 foci on Chr 17 was different in the two hybrids (Figure 3B). We evaluated the differences in MLH1 focus distributions, using two independent statistical tests. In the first test, the data (MLH1 focus positions) were grouped in 20 intervals, each 5% of SC length (about 350 nm for this chromosome), corresponding to the resolution limit of the analysis. After pooling intervals when sample sizes were too low, chi-square tests were applied to compare these distributions (see Materials and Methods). In the second test, Kolmogorov-Smirnov, we compared directly the distributions of the focus positions along the SC. Both analyses revealed a statistically significant difference of MLH1 distributions between B10xB10.A and RB2xB10.A hybrids (Table 2), indicating that the *Psmb9* control element affects CO distribution in several regions of Chr 17. In some intervals, the number of MLH1 foci was similar in both hybrids (for instance, intervals from 85% to 95% of SC length). In contrast, in other intervals, the RB2xB10.A hybrid displayed either a higher (region from 40% to 60% of SC length) or a lower density of MLH1 foci (region from 70% to 85% of SC length) as compared to B10xB10.A. At least two interpretations could be considered to account for these variations: either the unusually high recombination frequency at *Psmb9* leads to compensatory changes elsewhere on the chromosome or the *Psmb9* control element has independent effects in different regions of Chr 17.

**Table 2:**
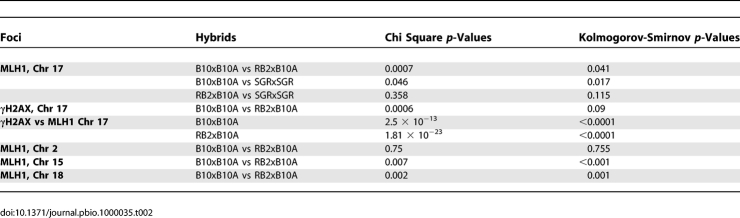
Statistical Analysis of MLH1 and γH2AX Foci Distributions

In order to distinguish between these possibilities, we mapped MLH1 foci along Chr 17 in SGRxSGR mice. In fact, these mice contain the genetic element–activating *Psmb9*; however, the *Psmb9* hotspot has low or no CO activity due to the presence of a repressive element located at the distal side of *Psmb9* on the SGR chromosome [38]. This was deduced from the analysis of B10xSGR and B10.AxSGR hybrids, and leads to the prediction that the same control should operate in the SGRxSGR mice analyzed here. In SGRxSGR mice, using the Kolmogorov-Smirnov test, we found that the MLH1 distribution was significantly different from that of B10xB10.A, but not from RB2xB10.A (Figure 3C, Table 2). In particular, the region 70%–85% showed a significant reduction of MLH1 foci in SGRxSGR compared to B10xB10.A (Fisher exact test, *p* = 0.00037). These results suggest that the redistribution of CO events along Chr 17 observed in RB2xB10A is not a consequence of the high activity at *Psmb9*, is not specific to the hybrid RB2xB10.A, and is rather due to the presence of the *wm7* regulatory element having multiple independent effects in several regions of Chr 17.

### The *wm7* Regulatory Element Influences DSB Formation in Several Regions on Chr 17

**Figure 4:**
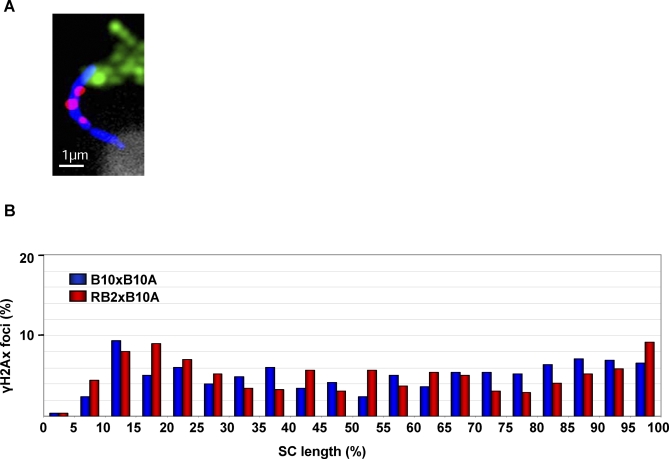
Different Distributions of γH2AX Foci on Chr 17 in B10xB10.A and RB2xB10.A (A) Chr 17 of a pachytene spermatocyte labeled with SYCP3 (blue), γH2AX (red), Chr 17 probe (mix of BACs RP23-10B20, and RP24-67L15; green), and stained with DAPI (white). (B) Percentage of γH2AX foci per 5% SC intervals on Chr 17 in B10xB10.A and RB2xB10.A hybrids (*n* = 549 and 513 foci, respectively).

**Figure 5:**
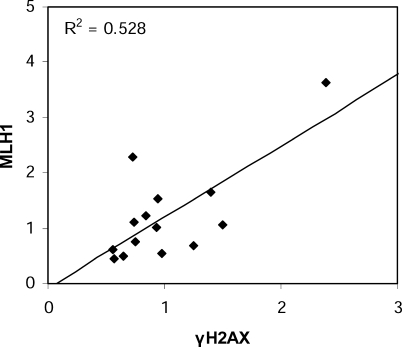
Correlation between Variations of γH2AX and MLH1 Distributions Due to the *Dsbc1* Locus The ratios between focus densities in RB2xB10.A and B10xB10.A hybrids per 5% Chr 17 SC length intervals (values of intervals 0%–25%, 25%–35%, and 35%–45% have been pooled) are represented for γH2AX (*x*-axis) and MLH1 (*y*-axis). The line resulting from the linear regression analysis between these ratios, and the coefficient of regression *R* ^2^, are shown.

The analysis of *cis* and *trans* regulations of *Psmb9* led to the conclusion that the high recombination activity at *Psmb9* is due to a regulation of the initiation rate [28,38]. In order to test whether the *wm7* regulatory element in RB2xB10.A modifies the frequency of initiation events along Chr 17 or only alters the choice of DSB repair towards COs or NCOs, we mapped γH2AX foci, the phosphorylated form of H2AX, along this chromosome. Among the markers that have been used to mark all meiotic DSB repair events, such as RAD51, DMC1, RPA, MSH4, and γH2AX, γH2AX gave the most reliable signals with our immuno-FISH protocol. γH2AX is detected as large chromosomal domains at leptotene and zygotene [44] and forms small foci on the SC at the beginning of pachytene [45,46]. The numbers of γH2AX foci, being in excess as compared to the number of MLH1 foci, are predicted to mark both CO- and NCO-designated events. This analysis shows an average number of 2.76 ± 0.17 and 2.67 ± 0.18 γH2AX foci per chromosome in B10xB10.A and RB2xB10.A hybrids, respectively. Interestingly, in both hybrids, the overall γH2AX distributions were significantly different from that of MLH1, with a larger fraction of foci on the proximal half of the chromosome (Figures 3–4, Table 2). This observation is similar to that described from the comparison of MSH4 or RPA and MLH1 foci and is thought to reflect specific CO regulations such as interference and crossover assurance [47]. In addition, the overall γH2AX distributions were significantly different in B10xB10.A and RB2xB10.A hybrids as determined by chi-square analysis (Table 2). Furthermore, regression analysis showed a significant correlation (*R* ^2^ = 0.528; *p* < 0.01) between the variations in MLH1 and γH2AX foci distributions in these two hybrids (Figure 5). These results strongly suggest that the *wm7* regulatory element is not simply inducing a redistribution of CO events on Chr 17 by regulating pathways of DSB repair but is influencing the formation of meiotic DSBs in several regions of this chromosome. We therefore named this genetic element, *Dsbc1* for Double-strand break control 1 and the two alleles *Dsbc1^wm7^* (present in the *wm7* haplotype) and *Dsbc1^b^* (B10 strain).

### *Dsbc1* Regulates Crossover Distribution on Other Chromosomes

**Figure 6:**
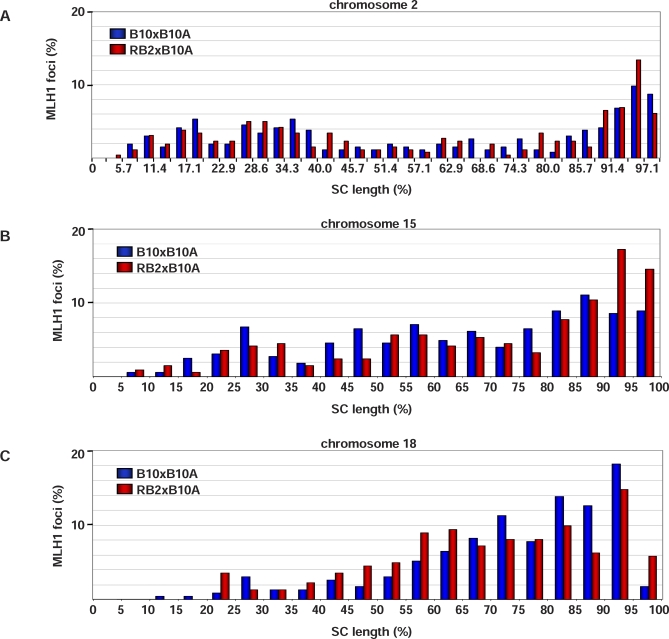
Distributions of MLH1 Foci on Different Chromosomes in B10xB10.A and RB2xB10.A Hybrids (A) Chr 2 divided into 35 intervals of 2.85% SC length (*n* = 269 and 263, respectively), (B) Chr 15 divided into 20 intervals of 5% SC length (*n* = 325 and 336, respectively), and (C) Chr 18 divided into 20 intervals of 5% SC length (*n* = 231 and 265, respectively).

As the presence of the *wm7* fragment leads to a redistribution of DSB and CO events over long chromosomal distances, we addressed the question of whether this regulation was specific to Chr 17 by monitoring CO distribution on other chromosomes. We thus analyzed MLH1 foci along Chrs 2, 15, and 18 in B10xB10.A and RB2xB10.A hybrids. For all three chromosomes, the total CO activity estimated by the average MLH1 number was identical in the two hybrids (Table S1). Using both chi-square and Kolmogorov-Smirnov tests, MLH1 distributions were found to be significantly different in B10xB10.A and RB2xB10.A on Chrs 15 and 18, showing that *Dsbc1* acts in *trans* on different chromosomes. We did not detect significant differences on Chr 2, however (Figure 6, Table 2).

### *Dsbc1^wm7^* Activates Recombination at the *Hlx1* Hotspot on Chr 1

**Figure 7:**
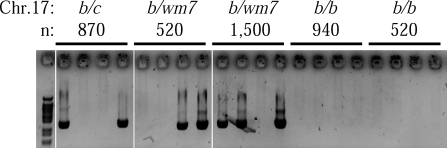
*Dsbc1* Controls CO Activity at the *Hlx1* Hotspot on Chr 1 COs (recombinant molecules B10-CAST) were detected at the *Hlx1* hotspot by allele-specific PCR on pools containing the indicated number (*n*) of amplifiable molecules, extracted from testes of 18-d-old mice of various genotypes for Chr 17 (*b/c*, Chr 17 of B10 and CAST origins; *b/wm7*, Chr 17 of B10 and R209 origins; *b/b*, both Chr 17 of B10 origin). The results from a subset of pools are shown. No recombinant molecules among 24,458 amplifiable genomes was observed in mice with the *b/b* genotype on Chr 17.

In order to show at the molecular level and at high resolution that *Dsbc1* regulates recombination activity on another chromosome, we tested the recombination activity at the *Hlx1* hotspot located on Chr 1 [30] in hybrids carrying the *wm7* haplotype on Chr 17. Parvanov et al. [39] have shown that the activity of this hotspot requires an allele from *Mus musculus castaneus* at a genetic locus located on Chr 17 and named *Rcr1* (see Discussion). Using an allele-specific PCR assay, we found a high CO activity at *Hlx1* in the presence of the *Dsbc1^wm7^* allele (for one type of recombinant molecule, *f*(CO) = 0.1 ± 0.07%) and no detectable CO in the presence of *Dsbc1^b^* (*f*(CO) < 0.015%; Figure 7). The frequencies of CO were found similar in the presence of *Dsbc1^wm7^* and in hybrids containing a Chr 17 from *M. m. castaneus*.

## Discussion

We report the identification of a novel regulatory element of the distribution of meiotic recombination events located between positions 10.1 and 16.8 Mb on mouse Chr 17. Comparative analysis between several hybrids suggests that a single locus, named *Dsbc1*, is regulating the distribution of crossing over in several regions of Chrs 15, 17, and 18. Two different alleles of *Dsbc1* appear to be present in B10 and mice derived from *M. m. molossinus*. Direct analysis of recombinant molecules shows that the *M. m. molossinus* allele of *Dsbc1*, *Dsbc1^wm7^*, stimulates the CO activity of two recombination hotspots, *Psmb9* on Chr 17 and *Hlx1* on Chr 1. We also demonstrate that *Dsbc1* controls the distribution of γH2AX foci along Chr 17. We thus propose that the *Dsbc1* locus controls the distribution of initiation of meiotic recombination genome wide in mice.

### The *Psmb9* Recombination Hotspot Is Regulated by a Distant Locus, *Dsbc1*

The *Psmb9* hotspot was known to be active in hybrids in which one parent carries the *wm7* haplotype from *M. m. molossinus*, the region of the *wm7* haplotype required for this activity extending from the centromere to the center of the hotspot. By genetic analysis, we show that the region necessary and sufficient for *Psmb9* activity is located between positions 10.1 and 16.8 Mb of Chr 17, at least 17.5 Mb away from *Psmb9*, indicating a long-distance control. Interestingly, the *Psmb9* hotspot was also found to be active in hybrids in which one parent carries the *cas3* MHC haplotype derived from *M. m. castaneus* [48]. This haplotype extends from the MHC to at least position 18.6 Mb towards the centromere (unpublished data) and therefore potentially overlaps with the regulatory element from the *wm7* haplotype. It is important to note that in most hybrids tested, *Dsbc1* is heterozygous, indicating a dominant or semidominant effect of the *Dsbc1^wm7^* allele over the *Dsbc1^b^*allele from the B10 strain. Heterozygosity is not required, however, since we have shown high *Psmb9* activity in hybrids homozygous at *Dsbc1^wm7^* [38].

### *Dsbc1* Controls Initiation of Meiotic Recombination

Based on measurements of CO and NCO frequencies, we infer that the enhanced recombination activity at *Psmb9* results from an enhancement of initiation frequency. The only alternative hypothesis would be to assume that B10 strain has a high initiation frequency at *Psmb9*, but that all DSBs are repaired using the sister chromatid, an unlikely hypothesis given the repression of sister-chromatid recombination during meiosis. Given that the presence of *Dsbc1^wm7^* on one homolog is sufficient to promote initiation on both [38], this locus acts, not only at long distance, but also in *trans*. A complementary approach to follow initiation events during meiosis is through the detection of γH2AX foci which mark meiotic DSBs [44]. Using this approach, we have shown that the distribution of γH2AX on Chr 17 is significantly different in B10xB10.A and RB2xB10.A hybrids, therefore in the absence or presence of the *Dsbc1^wm7^* allele. Although indirect, this observation is in agreement with the molecular analysis at *Psmb9*, and thus suggests that the *Dsbc1* locus controls the initiation of meiotic recombination in multiple genomic regions on Chr 17. Through the analysis of MLH1 focus distribution on Chr 17, we detect significant differences between the hybrids tested and found that the variations of γH2AX distribution are strongly correlated with those of MLH1. We therefore consider that these γH2AX variations are not likely due to modifications of the pathway leading to H2AX Ser129 phosphorylation that would be independent from DSB formation. We thus propose that *Dsbc1* regulates initiation activity and, therefore, CO rates. We do not exclude, however, that *Dsbc1* also affects other aspects of the recombination pathway after initiation.

### *Dsbc1* Acts Genome Wide

In order to test whether the *Dsbc1* locus acts on other chromosomes, we determined by immuno-FISH the localization of MLH1 foci on Chrs 2, 15, and 18. In all cases, the total CO activity as measured by the average number of MLH1 foci per chromosome was not changed. However, excepted for Chr 2, we found significant differences in the distribution of MLH1 foci along Chrs 15 and 18, depending on the presence or absence of the *Dsbc1^wm7^*allele. These differences are validated both by chi-square analysis after subdividing the data in intervals and by a comparison of the focus position distributions by the Kolmogorov-Smirnov test. In some regions, CO is increased, in others decreased, or not affected. The detection of these effects in single intervals is obviously limited by our assay in two respects: The spatial resolution and the statistical significance when sample size is small. The spatial resolution of MLH1 foci is close to 300–400 nm, corresponding to about 5 Mb or 1/20th of the chromosome length (Chrs 15, 17, and 18 are of similar lengths). If *Dsbc1* controls initiation as proposed above, it might do so independently at single sites or over domains, or both, and the net result over 5 Mb intervals is the sum of these effects. In the case of Chr 2, the number of foci measured per unit of physical distance was lower than on other chromosomes, potentially weakening the statistical significance of the measures. Pooling data to normalize for focus number per interval did not reveal significant differences, possibly due to decrease of spatial resolution. Within these constrains, it is not possible to determine which fraction of the hotspots are influenced by *Dsbc1*.

As a direct molecular demonstration that *Dsbc1* regulates, not only the *Psmb9* hotspot, but also hotspots on other chromosomes, we tested the *Hlx1* hotspot on Chr 1. We found that indeed the presence of *Dsbc1^wm7^* leads to a high CO frequency at *Hlx1*. This result not only demonstrates that *Dsbc1* acts on other chromosomes, but also that it might correspond to the same locus as one identified by Parvanov et al. [39]. In fact, the work by Parvanov et al. reports the identification of a locus, named *Rcr1*, whose position overlaps with the one of *Dsbc1* and which influences initiation activity at *Hlx1*. More specifically, the *Rcr1* allele from the Cast/Eij strain (*M. m. castaneus*) leads to a high CO frequency at *Hlx1* as well as variations of CO rates at several hotspots in the neighboring region of Chr 1. Genetically, although the mouse strains used in both approaches were different, it is interesting to note that *M. m. molossinus* is a hybrid between *M. m. domesticus* and *M. m. castaneus*. It is therefore possible that the *Dsbc1^wm7^*allele is derived from *M. m. castaneus*. Alternatively, the two loci *Rcr1* and *Dsbc1* might be distinct.

### How Does *Dsbc1* Act?

Our analysis reveals that the strains from B10 background and from *M. m. molossinus wm7* haplotype have two different alleles at the *Dsbc1* locus, *Dsbc1^b^* and *Dsbc1^wm7^*. Although the size of the genomic fragment from *wm7* origin is large and differs in several hybrids, the consistency of the effects leads us to assume that these are under the control of this single locus. The presence of one *Dsbc1^wm7^* allele is sufficient to induce hotspot activity at some sites (*Psmb9* and *Hlx1*, for instance). This allele thus behaves as a dominant trait specifying initiation at new locations. However, given that the total amount of recombination is constant (estimated by γH2AX and MLH1 foci), one expects the activity at other hotspots to decrease in the presence of *Dsbc1^wm7^* (heterozygous or homozygous). This could be due either to a regulation of the frequency of initiation events such as the one observed in S. cerevisiae in which DSBs can be suppressed by insertion of strong DSB sites nearby [19,49] or to a competition between *Dsbc1^wm7^* and *Dsbc1^b^* alleles. These observations raise several questions: Is the *Dsbc1^wm7^* allele an inducer of new initiation sites? Does the *Dsbc1^b^* play a role in initiation site specification? If so, which fractions of sites are controlled by the *Dsbc1* locus? At the molecular level, *Dsbc1* is expected to be a diffusible factor, RNA, or protein, that could act either on the recombination machinery involved in initiation or at the level of the substrate. One could, for instance, envision that *Dsbc1* controls directly or indirectly some aspects of chromatin accessibility that are known to be important for initiation site specification as shown by studies in yeast [50]. We have indeed obtained evidence that the structure of the chromatin at *Psmb9* differs in the context of *Dsbc1^wm7^* or *Dsbc1^b^* (J. Buard, P. Barthès, C. Grey, and B. de Massy, personal correspondence).

### How Such a Control Could Explain Previously Described Variations in Recombination Frequencies?

Modifiers of recombination have been described in fungi. In particular, in Neurospora crassa, three loci, rec-1, rec-2, and rec-3, have been found to influence meiotic recombination in a region-specific way and were proposed to modify patterns of initiation [51]. Recently, in Caenorhabditis elegans, mutations in a subunit of a condensing complex, DPY-28, were found to alter CO distribution by influencing DSB activity. Differently from the effect mediated by *Dsbc1*, dpy-28 mutants show an overall increase of CO frequencies [52]. However, such observations indicate that modifications in structural components of chromosomes can affect recombination initiation, and *Dsbc1* could therefore interact directly or indirectly with these components.

Genetic control of the frequency of meiotic recombination has been suggested from analysis of several hotspots in the mouse genome. For instance, a very active hotspot was identified in the *Eβ* gene, for which activity was suppressed in the presence of the *p* haplotype [53,54]. Although the *Eβ* hotspot is active in many different genetic contexts, its activity is highly variable in different hybrids [29,54]. The activity of the *Eβ* hotspot was suggested to depend on distal elements or to be influenced by genetic background [34]. Among other less-characterized hotspots some are haplotype-specific such as the *Pb* hotspot, active in the presence of the *cas4* haplotype derived from *M. m. castaneus* [55,56], whereas others, such as *G7c*, have detectable activity in a variety of hybrids [57]. Whether these variations depend on local or distal controls is not known. In humans, the regulation of CO activity by remote control has been proposed from the analysis of the *MSTM1a* hotspot showing variable CO frequencies in men with identical haplotypes in the 100-kb region around the hotspot [58]. In addition, genome-wide mapping has shown a heritable variation in hotspot activity between individuals [33]; it would be interesting to know whether specific loci are responsible for such effects and whether any locates to the region of the human genome synthenic to that of *Dsbc1*.

### Implications for Hotspot Evolution

If a single locus can modify initiation activity in such a dramatic way as we have uncovered, it obviously has consequences, not only on genetic maps as mentioned above, but also on their evolution. In particular, it provides an answer to the hotspot paradox. In fact, due to the directionality of mismatch repair during DSB repair in meiosis, the initiating chromatid is the recipient of genetic information. Consequently, in a population, if an allele appears that lowers initiation frequency in *cis*, and is located in the region subject to gene conversion, its frequency will tend to increase, and it will eventually be fixed. This phenomenon has been called the hotspot paradox because it is expected to lead to loss of hotspot activity in the genome and to strongly limit the appearance of new hotspots [59]. In fact, we have already reported that at the *Psmb9* hotspot, a suppressive mutation, as observed in the SGR strain, might have spread rapidly through the population due to the disparity of gene conversion and the very high efficiency of the *Psmb9* site in this context [38]. The forces that maintain hotspot activity remain unknown. If the activity of a hotspot can be influenced by elements outside the region that is subject to gene conversion, alleles that lower initiation frequency might not necessarily be driven to fixation, and new hotspots could appear independently from mutations in the region of the hotspot itself [60]. We propose that elements such as *Dsbc1* that regulate in *trans* the distribution of recombination sites can counteract the loss of activity at some sites. If true, this suggests that the *Dsbc1* locus itself might be under specific selection to maintain the recombination activity at an appropriate level in the genome.

## Materials and Methods

### Mouse strains.

The mouse strains used in this study were C57BL/10JCrl (B10), B10.A, B10.MOL-SGR (SGR), B10.A(R209) (R209), and CAST/EiJ (CAST). The strains R5, RJ3, and RB2 have been obtained by back-crossing B10 x R209 F1 with B10 and screening the progeny for CO events between *D17Mit164* and the *Psmb9* hotspot. The recombinant Chr 17 of strains RJ2 and RK2 have been generated in a RB2xB10.A F1 and a RB2xB10 F1 hybrid, respectively. The recombinant Chr 17 of strain R115 has been generated in a *Mlh1^tm1Lisk^* × R209 F1 hybrid, backcrossed with R209. For the molecular analysis at the *Hlx1* hotspot, mice heterozygous for the B10 and CAST alleles at the chromosome 1 *Hlx1* hotspot and carrying the appropriate genotype on Chr 17 have been generated as follows: CASTxR209 F1 hybrids were generated and crossed with B10. Males heterozygous at the *Hlx1* hotspot and containing either the haplotypes from B10 and CAST (*b/c*) or the haplotypes from B10 and R209 (*b/wm7*) on Chr 17, were analyzed. In addition, *b/c* females were crossed with B10 in order to generate males heterozygous at the *Hlx1* hotspot and homozygous *b/b* on Chr 17. The region of Chr 17 taken into account in this analysis is the proximal third, from *D17Mit164* (position 3.9 Mb) to *D17Mit21* (position 34.4 Mb).

### Microsatellite mapping.

The markers used for mapping the fragments derived from R209 in the recombinant lines are listed in Table S2. Their position on Chr 17 sequence is based on the National Center for Biotechnology Information (NCBI) m37 mouse assembly. The sequences of the primers used for amplifying the microsatellite markers have been found on the Mouse Genome Informatics site (http://www.informatics.jax.org/). The PCR cycling conditions were 94 °C for 10 s, 55 °C for 30 s, and 72 °C for 30 s for 36 cycles.

### Detection of COs and NCOs.

The allele-specific PCR protocol for the direct molecular detection and analysis of recombination products at *Psmb9* and at *Hlx1* in sperm or testis DNA was as described [38,61]. For *Hlx1*, the cycling conditions of the first PCR using the primer pair BF1/CR1 were: denaturation at 94 °C for 2 min followed by 25 cycles at 94 °C for 15 s, 64 °C for 30 s, 68 °C for 210 s, and 68 °C for 5 min as the final extension step. The cycling conditions of the second PCR using the primer pair BF2/CR2 were: Denaturation at 94 °C for 2 min followed by 28 cycles at 94 °C for 15 s, 61 °C for 30 s, 68 °C for 210 s, and 68 °C for 5 min as the final extension step. The primers used for the detection of COs and NCOs are listed in Table S3.

### Nuclei spreads.

Spermatocyte nuclei spreads were performed by the dry-down technique as described [62].

### Probes for fluorescence in situ hybridization (FISH).

Chromosome-specific BAC probes were labeled with FISH Tag DNA Kit (Invitrogen) according to the manufacturer's manual. BAC probes were selected to be between 120 and 220 kb in size and poor in repetitive elements. On Chr 17, for colocalization assays, analysis of focus distribution, and SC length measurements, we used two BACs (RP24-67L15 and RP23-10B20) that locate at 86.3 Mb and 87.6 Mb, respectively, on Chr 17 and were mixed together. To determine the position of *Psmb9* on SCs, one BAC probe that covers the *Psmb9* sequence was used (RP23-95J18). To determine the global SC structure, we used two different BAC probes (RP23-268M4 and RP24-279K24) that locate at 46.2 Mb and 73.4 Mb, respectively. For Chrs 2, 15, and 18, we used single BAC probes (RP23-101G16, RP23-408L3 and RP24-266A12, respectively). To identify the *Psmb9* hotspot, six DNA fragments of 800 to 1,200 bp covering a region of 10 kb around *Psmb9* were amplified by PCR (P1U: 5′-CCCCTTCCTGTAGACAT-3′, P1L: 5′-ACAAATAAGCATATACCACG-3′, P2U: 5′-GCCATGTTATTTCTTGACATG-3′, P2L: 5′-CCACACAGGATAAATAATGCT-3′, P3U: 5′-AAATTAAAAAGTCAACCC-3′, P3L: 5′-AGCTGGAGTTACAGGTGTT-3′, P4U: 5′-CTCTGGACCACAAAGCTAGAA-3′, P4L: 5′-CAGAGCCAAGCACATCTAACT-3′, P5U: 5′-GCAACGGTGGTTGTATGG-3′, P5L: 5′-GAAGGTGTGGGGGAAGTAGAT-3′, P6U: 5′-GAATGAGCTTCCCAAGTTGAC-3′, and P6L: 5′-CCCTGGCCTGTCGTGTT-3′). An equimolar mix of all PCR fragments was biotinylated with the BioNick Labeling System (Invitrogen) according to the manufacturer's manual.

### In situ hybridization.

For the FISH, we adapted the protocol of H. Scherthan [63] to our purpose. Briefly, spermatocyte spreads were treated with NaSCN (1 M) at 70 °C for 30 min; washed in 1× sodium saline citrate (SSC) solution, and digested with RNAse A (100 μg/ml) at 37 °C for 1 h. The slides were then washed with 1× SSC solution, covered with a solution of 70% formamide/2× SSC, and the DNA was denatured by heating at 85 °C for 5 min. After denaturation, the slides were immediately washed in ice-cold 2× SSC. Probes (300 ng per slide for *Psmb9* PCR probe or 80 ng per slide for BAC probes) were denatured in 10–15 μl of hybridization solution (50% formamide, 2× SSC solution, 10% dextran, and mouse Cot1-DNA 10 ng/μl) for 3 min at 93 °C. Hybridization solution was applied to slides, covered with a cover slip, sealed with rubber cement, and incubated in a humid chamber at 37 °C for 24 h. The slides were then washed in 0.05× SSC at 37 °C and treated for immunostaining.

### Immunostaining.

Immunostaining was performed as described [64], using a milk-based blocking buffer (5% milk, 5% donkey serum in 1× PBS, phosphate buffer saline). Antibodies were guinea pig anti-SYCP3 at 1:500 dilution, mouse monoclonal anti-MLH1 (Pharmigen) at 1:50 dilution, and mouse monoclonal anti–phospho-H2A.X (Upstate) at 1:20,000. All incubations with primary antibodies were performed overnight at room temperature. Secondary antibodies were goat anti–guinea pig Alexa Fluor 488 (Molecular Probes) and donkey Cy3-conjugated anti-mouse. Incubations with secondary antibodies were performed at 37 °C for 1.5 h. *Psmb9* PCR probe was revealed with Cy5-conjugated Streptavidin (Jackson Immuno Research) at 1:750 dilution, incubation was performed at 37 °C for 1 h. Incubations with secondary antibodies were performed simultaneously, whereas incubation with Cy5-conjugated Streptavidin was always preformed last. Nuclei were stained with DAPI (4′-6-Diamidino-2-phenylindole, 2 μg/ml) during the final washing step.

### Fluorescence microscopy image acquisition and analysis.

Digital images were obtained by using a cooled charge-coupled device (CCD) camera, Coolsnap HQ (Photometrics) coupled to a Leica DM 6000B microscope. Each color signal was acquired as a black-and-white image using appropriate filter sets and was merged with Metamorph Imaging software.

### Analysis of MLH1/*Psmb9* colocalization on SCs.

The identity of Chr 17 was determined by the use of a mix of two Chr 17–specific BAC probes (RP24-67L15 and RP23-10B20). To asses the colocalization frequency, only nuclei that were MLH1 positive on Chr 17 were counted. Distances between the center of MLH1 and *Psmb9* foci were measured with Metamorph Imaging software. Colocalization was considered when the distance between the centers of both foci (MLH1 and *Psmb9*) was less than 300 nm (4.6 pixels with a 100× lens).

### Analysis of SC length, MLH1, and γH2AX distributions.

The chromosome to be analyzed was identified using a BAC probe that was revealed with a different color than the SC. SC length was measured with Metamorph Imaging software. To determine the distribution of MLH1 (or γH2AX) foci, the position of each focus on the chromosome was recorded as a relative distance (percentage of total SC length) from the centromere. The centromeric end was identified by surrounding DAPI-bright heterochromatin. MLH1 (or γH2AX) foci were mapped when MLH1 (or γH2AX) and SYCP3 signals overlapped.

### Statistical analysis.

Distributions were compared both by chi-square analysis and by the nonparametric Kolmogorov-Smirnov test. For chi-square analysis, all focus positions were grouped in 20 intervals of 5% SC length (about 5 Mb) for Chrs 15, 17, and 18 and in 35 intervals of 2.85% SC length (about 5 Mb) for Chr 2 (Tables S4, S5, and S6). Interval contents were systematically examined starting from the centromere proximal end for each chromosome. If necessary, intervals were pooled such as to have expected sample sizes of five foci minimum. For each chromosome and each hybrid, data were obtained from two different mice (except for Chr 2, one mouse, and for MLH1 analysis on Chr 17 in RB2xB10.A, four mice). In each case, the homogeneity of the two sets of data was validated by chi-square and Kolmogorov-Smirnov analysis showing the reproducibility of distributions for a given genotype on a given chromosome (Table S7). The Kolmogorov-Smirnov test compares the distribution of foci positions on the SC and quantifies the distance between two distributions. For the analysis of variations of γH2AX and MLH1 focus densities on Chr 17, the ratios between focus percentages per 5% SC length intervals in RB2xB10.A and in B10xB10.A were calculated (values of intervals 0%–25%, 25%–35%, and 35%–45% were pooled due to the small number of MLH1 foci in these regions), for each type of foci (γH2AX and MLH1). A linear regression analysis was performed to compare the variations of γH2AX and MLH1 focus densities.

## Supporting Information

Figure S1 Percentage of Nuclei with One, Two, Three, or Four *Psmb9* Foci in B10xB10.A and RB2xB10.A The RB2xB10.A hybrid (*n* = 316) has a single focus significantly more often than B10xB10.A (*n* = 275) has, (Fisher exact test, *p*-value < 0.00001).
(21 KB PDF)

Figure S2 Position of *Psmb9* Is Around 50% of Whole Chr 17 SC Length and Is Not Significantly Different in B10xB10.A and RB2xB10.A Hybrids (A) Position of *Psmb9* was assessed by measuring the relative position of the *Psmb9* BAC probe (red) on SC of Chr 17 in spermatocyte spreads stained with anti-SYCP3 (blue) and DAPI (white).
(B) Average position is 48.2 ± 1.5% and 47.9 ± 1.6% of total SC length respectively. 83.9% of *Psmb9* BAC probes in B10xB10.A hybrids (*n* = 62) and 81.6% in RB2xB10.A (*n* = 49) are situated in the interval of 40%–55% of total SC length. Similar results were obtained when the *Psmb9* PCR probe was used (unpublished data).
(34 KB PDF)

Table S1 SC Lengths and Average MLH1 Focus Number on Chrs 2, 15, and 18 (22 KB PDF)

Table S2 Microsatellite Markers (33 KB PDF)

Table S3 Sequences of the Primers Used for the Detection of COs and NCOs (26 KB PDF)

Table S4 Number of MLH1 Foci per Intervals on Chrs 15, 17, and 18 (15 KB PDF)

Table S5 Number of MLH1 Foci per Intervals on Chr 2 (9 KB PDF)

Table S6 Number of γH2AX Foci per Intervals on Chr 17 (35 KB PDF)

Table S7 Intragenotype Statistical Analysis of MLH1 and γH2AX Foci Distributions (17 KB PDF)

## Abbreviations

BAC: bacterial artificial chromosome
Chr: Chromosome
CO: crossover
DSB: DNA double-strand break
FISH: fluorescent in situ hybridization
NCO: noncrossover
SC: synaptonemal complex

## References

[pbio-1000035-b001] Petronczki M, Siomos MF, Nasmyth K (2003). Un menage a quatre. The molecular biology of chromosome segregation in meiosis. Cell.

[pbio-1000035-b002] Marais G, Charlesworth B (2003). Genome evolution: recombination speeds up adaptive evolution. Curr Biol.

[pbio-1000035-b003] Hunter N, Aguilera A, Rothstein R (2007). Meiotic recombination. Molecular genetics of recombination.

[pbio-1000035-b004] Keeney S (2001). Mechanism and control of meiotic recombination initiation. Curr Top Dev Biol.

[pbio-1000035-b005] Baudat F, de Massy B (2007). Regulating double-stranded DNA break repair towards crossover or non-crossover during mammalian meiosis. Chromosome Res.

[pbio-1000035-b006] Lynn A, Soucek R, Borner GV (2007). ZMM proteins during meiosis: crossover artists at work. Chromosome Res.

[pbio-1000035-b007] Baudat F, Nicolas A (1997). Clustering of meiotic double-strand breaks on yeast chromosome III. Proc Natl Acad Sci U S A.

[pbio-1000035-b008] Gerton JL, DeRisi J, Shroff R, Lichten M, Brown PO (2000). Inaugural article: global mapping of meiotic recombination hotspots and coldspots in the yeast Saccharomyces cerevisiae. Proc Natl Acad Sci U S A.

[pbio-1000035-b009] Nicolas A (1998). Relationship between transcription and initiation of meiotic recombination: toward chromatin accessibility. Proc Natl Acad Sci U S A.

[pbio-1000035-b010] Ohta K, Shibata T, Nicolas A (1994). Changes in chromatin structure at recombination initiation sites during yeast meiosis. EMBO J.

[pbio-1000035-b011] Wu T-C, Lichten M (1994). Meiosis-induced double-strand break sites determined by yeast chromatin structure. Science.

[pbio-1000035-b012] Borde V, Wu TC, Lichten M (1999). Use of a recombination reporter insert to define meiotic recombination domains on chromosome III of Saccharomyces cerevisiae. Mol Cell Biol.

[pbio-1000035-b013] Mizuno K, Emura Y, Baur M, Kohli J, Ohta K (1997). The meiotic recombination hot spot created by the single- base substitution ade6-M26 results in remodeling of chromatin structure in fission yeast. Genes Dev.

[pbio-1000035-b014] Mizuno K, Hasemi T, Ubukata T, Yamada T, Lehmann E (2001). Counteracting regulation of chromatin remodeling at a fission yeast cAMP response element-related recombination hotspot by stress-activated protein kinase, cAMP-dependent kinase and meiosis regulators. Genetics.

[pbio-1000035-b015] Steiner WW, Schreckhise RW, Smith GR (2002). Meiotic DNA breaks at the S. pombe recombination hot spot M26. Mol Cell.

[pbio-1000035-b016] Hirota K, Steiner WW, Shibata T, Ohta K (2007). Multiple modes of chromatin configuration at natural meiotic recombination hot spots in fission yeast. Eukaryot Cell.

[pbio-1000035-b017] Cromie GA, Hyppa RW, Cam HP, Farah JA, Grewal SI (2007). A discrete class of intergenic dna dictates meiotic DNA break hotspots in fission yeast. PLoS Genet.

[pbio-1000035-b018] Pecina A, Smith KN, Mezard C, Murakami H, Ohta K (2002). Targeted stimulation of meiotic recombination. Cell.

[pbio-1000035-b019] Robine N, Uematsu N, Amiot F, Gidrol X, Barillot E (2007). Genome-wide redistribution of meiotic double-strand breaks in Saccharomyces cerevisiae. Mol Cell Biol.

[pbio-1000035-b020] Arnheim N, Calabrese P, Tiemann-Boege I (2007). Mammalian meiotic recombination hot spots. Annu Rev Genet.

[pbio-1000035-b021] Buard J, de Massy B (2007). Playing hide and seek with mammalian meiotic crossover hotspots. Trends Genet.

[pbio-1000035-b022] McVean GA, Myers SR, Hunt S, Deloukas P, Bentley DR (2004). The fine-scale structure of recombination rate variation in the human genome. Science.

[pbio-1000035-b023] Myers S, Bottolo L, Freeman C, McVean G, Donnelly P (2005). A fine-scale map of recombination rates and hotspots across the human genome. Science.

[pbio-1000035-b024] Myers S, Freeman C, Auton A, Donnelly P, McVean G (2008). A common sequence motif associated with recombination hot spots and genome instability in humans. Nat Genet.

[pbio-1000035-b025] Jensen-Seaman MI, Furey TS, Payseur BA, Lu Y, Roskin KM (2004). Comparative recombination rates in the rat, mouse, and human genomes. Genome Res.

[pbio-1000035-b026] Kong A, Gudbjartsson DF, Sainz J, Jonsdottir GM, Gudjonsson SA (2002). A high-resolution recombination map of the human genome. Nat Genet.

[pbio-1000035-b027] Nachman MW, Churchill GA (1996). Heterogeneity in rates of recombination across the mouse genome. Genetics.

[pbio-1000035-b028] Guillon H, de Massy B (2002). An initiation site for meiotic crossing-over and gene conversion in the mouse. Nat Genet.

[pbio-1000035-b029] Yauk CL, Bois PR, Jeffreys AJ (2003). High-resolution sperm typing of meiotic recombination in the mouse MHC E(beta) gene. EMBO J.

[pbio-1000035-b030] Paigen K, Szatkiewicz JP, Sawyer K, Leahy N, Parvanov ED (2008). The recombinational anatomy of a mouse chromosome. PLoS Genet.

[pbio-1000035-b031] Lynn A, Ashley T, Hassold T (2004). Variation in human meiotic recombination. Annu Rev Genomics Hum Genet.

[pbio-1000035-b032] Heine D, Khambata S, Passmore HC (1998). High-resolution mapping and recombination interval analysis of mouse chromosome 17. Mamm Genome.

[pbio-1000035-b033] Coop G, Wen X, Ober C, Pritchard JK, Przeworski M (2008). High-resolution mapping of crossovers reveals extensive variation in fine-scale recombination patterns among humans. Science.

[pbio-1000035-b034] Heine D, Khambata S, Wydner KS, Passmore HC (1994). Analysis of recombinational hot spots associated with the p haplotype of the mouse MHC. Genomics.

[pbio-1000035-b035] Khambata S, Mody J, Modzelewski A, Heine D, Passmore HC (1996). Ea recombinational hot spot in the mouse major histocompatibility complex maps to the fourth intron of the Ea gene. Genome Res.

[pbio-1000035-b036] Shiroishi T, Hanzawa N, Sagai T, Ishiura M, Gojobori T (1990). Recombinational hotspot specific to female meiosis in the mouse major histocompatibility complex. Immunogenetics.

[pbio-1000035-b037] Shiroishi T, Sagai T, Hanzawa N, Gotoh H, Moriwaki K (1991). Genetic control of sex-dependent meiotic recombination in the major histocompatibility complex of the mouse. EMBO J.

[pbio-1000035-b038] Baudat F, de Massy B (2007). Cis- and trans-acting elements regulate the mouse Psmb9 meiotic recombination hotspot. PLoS Genet.

[pbio-1000035-b039] Parvanov ED, Ng SGS, Petkov PM, Paigen K (2009). Trans-regulation of mouse meiotic recombination hotspots by Rcr1. PLoS Biol.

[pbio-1000035-b040] Shiroishi T, Sagai T, Moriwaki K (1982). A new wild-derived H-2 haplotype enhancing K-IA recombination. Nature.

[pbio-1000035-b041] Yoshino M, Sagai T, Lindahl KF, Toyoda Y, Moriwaki K (1995). Allele-dependent recombination frequency: homology requirement in meiotic recombination at the hot spot in the mouse major histocompatibility complex. Genomics.

[pbio-1000035-b042] Marcon E, Moens P (2003). MLH1p and MLH3p localize to precociously induced chiasmata of okadaic-acid-treated mouse spermatocytes. Genetics.

[pbio-1000035-b043] Froenicke L, Anderson LK, Wienberg J, Ashley T (2002). Male mouse recombination maps for each autosome identified by chromosome painting. Am J Hum Genet.

[pbio-1000035-b044] Mahadevaiah SK, Turner JM, Baudat F, Rogakou EP, de Boer P (2001). Recombinational DNA double-strand breaks in mice precede synapsis. Nat Genet.

[pbio-1000035-b045] Chicheportiche A, Bernardino-Sgherri J, de Massy B, Dutrillaux B (2007). Characterization of Spo11-dependent and independent phospho-H2AX foci during meiotic prophase I in the male mouse. J Cell Sci.

[pbio-1000035-b046] Lenzi ML, Smith J, Snowden T, Kim M, Fishel R (2005). Extreme heterogeneity in the molecular events leading to the establishment of chiasmata during meiosis i in human oocytes. Am J Hum Genet.

[pbio-1000035-b047] de Boer E, Stam P, Dietrich AJ, Pastink A, Heyting C (2006). Two levels of interference in mouse meiotic recombination. Proc Natl Acad Sci U S A.

[pbio-1000035-b048] Uematsu Y, Kiefer H, Schulze R, Fischer-Lindahl K, Steinmetz M (1986). Molecular characterization of a meiotic recombinational hotspot enhancing homologous equal crossing-over. EMBO J.

[pbio-1000035-b049] Jessop L, Allers T, Lichten M (2005). Infrequent co-conversion of markers flanking a meiotic recombination initiation site in Saccharomyces cerevisiae. Genetics.

[pbio-1000035-b050] Lichten M, Lankenau DH (2008). Meiotic chromatin: the substrate for recombination initiation. Genome dynamics and stability.

[pbio-1000035-b051] Whitehouse HLK, Whitehouse HLK (1982). Recombination in eucaryotes. Genetic recombination: understanding the mechanisms.

[pbio-1000035-b052] Tsai CJ, Mets DG, Albrecht MR, Nix P, Chan A (2008). Meiotic crossover number and distribution are regulated by a dosage compensation protein that resembles a condensin subunit. Genes Dev.

[pbio-1000035-b053] Lafuse WP, David CS (1986). Recombination hot spots within the I region of the mouse H-2 complex map to the E beta and E alpha genes. Immunogenetics.

[pbio-1000035-b054] Steinmetz M, Minard K, Horvath S, McNicholas J, Srelinger J (1982). A molecular map of the immune response region from the major histocompatibility complex of the mouse. Nature.

[pbio-1000035-b055] Isobe T, Yoshino M, Mizuno K, Lindahl K, Koide T (2002). Molecular characterization of the Pb recombination hotspot in the mouse major histocompatibility complex class II region. Genomics.

[pbio-1000035-b056] Steinmetz M, Stephan D, Fischer Lindahl K (1986). Gene organization and recombinational hotspots in the murine major histocompatibility complex. Cell.

[pbio-1000035-b057] Snoek M, Teuscher C, van Vugt H (1998). Molecular analysis of the major MHC recombinational hot spot located within the G7c gene of the murine class III region that is involved in disease susceptibility. J Immunol.

[pbio-1000035-b058] Neumann R, Jeffreys AJ (2006). Polymorphism in the activity of human crossover hotspots independent of local DNA sequence variation. Hum Mol Genet.

[pbio-1000035-b059] Boulton A, Myers RS, Redfield RJ (1997). The hotspot conversion paradox and the evolution of meiotic recombination. Proc Natl Acad Sci U S A.

[pbio-1000035-b060] Peters AD (2008). A combination of cis and trans control can solve the hotspot conversion paradox. Genetics.

[pbio-1000035-b061] Guillon H, Baudat F, Grey C, Liskay RM, de Massy B (2005). Crossover and noncrossover pathways in mouse meiosis. Mol Cell.

[pbio-1000035-b062] Peters A, Plug AW, vanVugt MJ, deBoer P (1997). Drying-down technique for the spreading of mammalian meiocytes from the male and female germline. Chromosome Res.

[pbio-1000035-b063] Scherthan H (2002). Detection of chromosome ends by telomere FISH. Methods Mol Biol.

[pbio-1000035-b064] Moens PB, Chen DJ, Shen ZY, Kolas N, Tarsounas M (1997). Rad51 immunocytology in rat and mouse spermatocytes and oocytes. Chromosoma.

