# Supplementary material for: Genome-Wide Control of the Distribution of Meiotic Recombination
Source: PLoS Biol. 2009 Feb 17;7(2):e1000035. doi: 10.1371/journal.pbio.1000035 (PMC2642883; doi:10.1371/journal.pbio.1000035)
Supplement: Supplementary file 1 [file pbio.1000035.sg001.pdf]

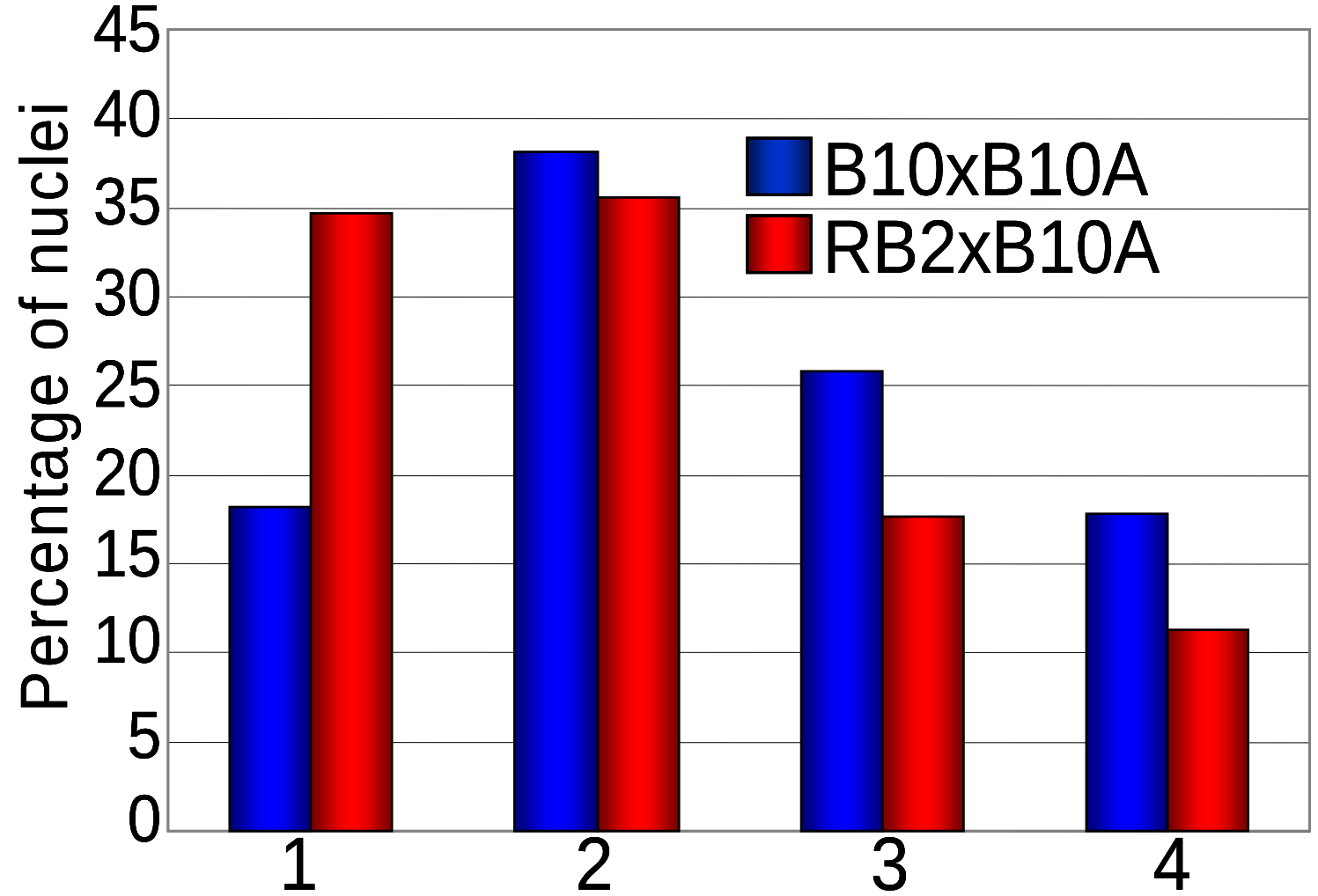
Percentage of nuclei
45
40
35
30
25
20
15
10
5
0
1
2
3
4
B10xB10A
RB2xB10A
