# Supplementary material for: Genome-Wide Control of the Distribution of Meiotic Recombination
Source: PLoS Biol. 2009 Feb 17;7(2):e1000035. doi: 10.1371/journal.pbio.1000035 (PMC2642883; doi:10.1371/journal.pbio.1000035)
Supplement: Supplementary file 2 [file pbio.1000035.sg002.pdf]

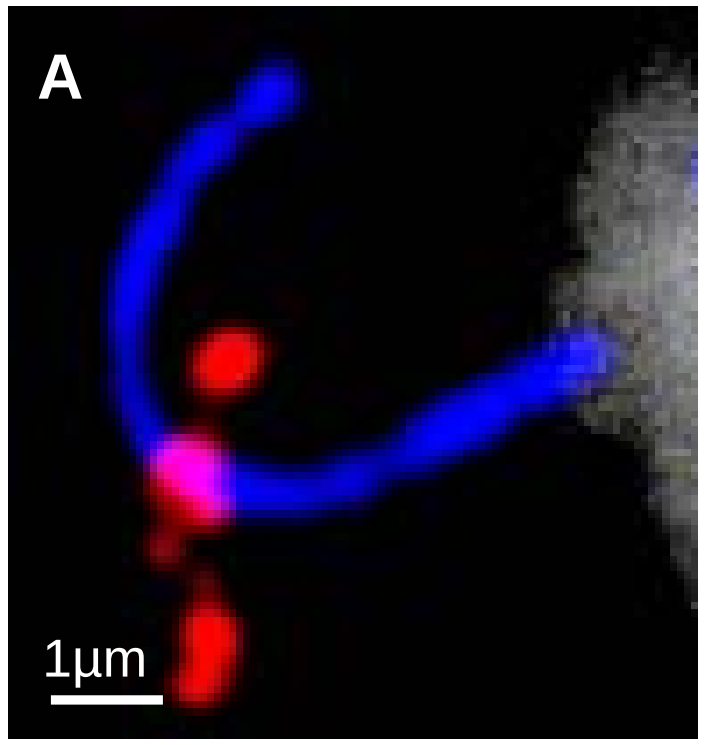
A
1µm

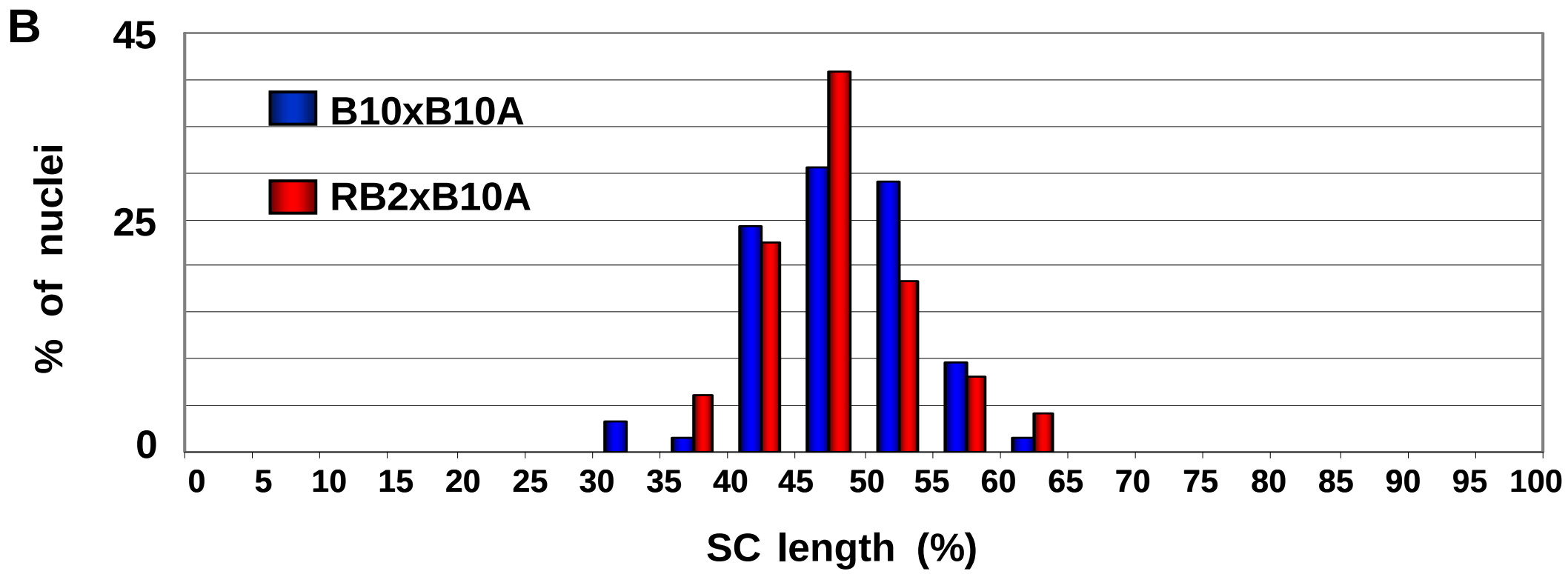
B
45
25
0
% of nuclei
B10xB10A
RB2xB10A
0
5
10
15
20
25
30
35
40
45
50
55
60
65
70
75
80
85
90
95
100
SC length (%)
