# Supplementary material for: Genome-Wide Control of the Distribution of Meiotic Recombination
Source: PLoS Biol. 2009 Feb 17;7(2):e1000035. doi: 10.1371/journal.pbio.1000035 (PMC2642883; doi:10.1371/journal.pbio.1000035)
Supplement: Supplementary file 3 [file pbio.1000035.st001.pdf]

Table S1

chromosome 2	B10xB10A	RB2xB10A
SC length (μm)	12.72 ± 0.25	13.08 ± 0.28
average Mlh1 focus number	1.67 ± 0.07	1.65 ± 0.07
Number of foci	269	263
Number of nuclei	161	159

chromosome 15	B10xB10A	RB2xB10A
SC length (μm)	7.40 ± 0.13	7.03 ± 0.12
average Mlh1 focus number	1.08 ± 0.03	1.08 ± 0.03
Number of foci	325	336
Number of nuclei	301	311

chromosome 18	B10xB10A	RB2xB10A
SC length (μm)	6.71 ± 0.17	5.91 ± 0.10
average Mlh1 focus number	0.99 ± 0.02	0.99 ± 0.02
Number of foci	231	265
Number of nuclei	234	267
