# Supplementary material for: Genome-Wide Control of the Distribution of Meiotic Recombination
Source: PLoS Biol. 2009 Feb 17;7(2):e1000035. doi: 10.1371/journal.pbio.1000035 (PMC2642883; doi:10.1371/journal.pbio.1000035)
Supplement: Supplementary file 4 [file pbio.1000035.st002.pdf]

Table S2: Mapping of the fragments derived from *M. m. molossinus* in recombinant strains.

Marker	Position (Mb) NCBI m37 mouse assembly	R209	R5	RJ3	R115	RB2	RJ2	RK2
D17Mit164	3.925	*wm7*	*b*	*b*	*b*	*wm7*	*b*	*wm7*
D17Mit19	4.804	*wm7*	*b*	*b*	*b*	*wm7*	*b*	*wm7*
D17Mit143	8.636	*wm7*	*wm7*	*b*	*b*	*wm7*	*b*	*wm7*
D17Mit57	10.055	*wm7*	*wm7*	*b*	*b*	*wm7*	*b*	*wm7*
D17Mit58	10.489	*wm7*	*wm7*	*b*	n.d.	*wm7*	*wm7*	*wm7*
D17Mit113	12.172	*wm7*	*wm7*	*b*	*b*	*wm7*	*wm7*	*wm7*
D17Mit114	12.611	*wm7*	*wm7*	*b*	*b*	*wm7*	*wm7*	n. d.
D17Mit222	13.377<br>13.660	*wm7*	n. d.	n. d.	*b*	*wm7*	*wm7*	*wm7*
D17Mit213	16.752	*wm7*	*wm7*	*b*	*b*	*wm7*	*wm7*	*b*
D17Mit59	18.640	*wm7*	n. d.	*b*	*b*	*wm7*	*wm7*	*b*
D17Mit197	20.265	*wm7*	*wm7*	*b*	*b*	*wm7*	*wm7*	n. d.
D17Jcs99	14.053<br>21.181	*wm7*	n. d.	n. d.	*b*	*wm7*	n. d.	n. d.
D17Jcs11	24.114	*wm7*	n. d.	n. d.	*wm7*	*wm7*	*wm7*	n. d.
D17Mit133	24.995	*wm7*	*wm7*	*b*	*wm7*	*b*	*b*	n. d.
D17Mit30	27.634	*wm7*	*wm7*	*b*	*wm7*	*b*	*b*	n. d.
D17Mit198	27.796	*wm7*	*wm7*	*b*	*wm7*	*b*	*b*	*b*
D17Mit28	34.138	*wm7*	*wm7*	*wm7*	*wm7*	*b*	*b*	n. d.
Psmb9	34.317	*wm7-a*	*wm7-a*	*wm7-a*	*wm7-a*	*b*	*b*	*b*
