# Supplementary material for: Genome-Wide Control of the Distribution of Meiotic Recombination
Source: PLoS Biol. 2009 Feb 17;7(2):e1000035. doi: 10.1371/journal.pbio.1000035 (PMC2642883; doi:10.1371/journal.pbio.1000035)
Supplement: Supplementary file 5 [file pbio.1000035.st003.pdf]

Table S3: Primers used for the detection of CO and NCO

Name	Sequence	Locus	PCR	CO/NCO	Strain specificity
269U20	TTAGCTTCTGAGCCATCTGT	*Psmb9*	1st	CO, NCO	B10, RB2, RJ2
269U20b	TCAACTTCTGAGCCGTCTGA	*Psmb9*	1st	CO, NCO	B10.A, R209, R5, RJ3, R115
4623L20	ATTTGAGAGTGGGAGGGGAT	*Psmb9*	1st	CO, NCO	B10, RB2, RJ2
4623L20b	ATTTGAGAGTGAGAGGGGAC	*Psmb9*	1st	CO, NCO	B10.A, R209, R5, RJ3, R115
402U19	CTTCCTCTTGCCATTACCG	*Psmb9*	2nd	CO	B10, RB2, RJ2
402U19b	CCTCCTCCTACCATTACCT	*Psmb9*	2nd	CO	B10.A, R209, R5, RJ3, R115
3402L20	AATAAAGGGCTAGCTGTGCA	*Psmb9*	2nd	CO, NCO	B10, RB2, RJ2
3402L20b	AATAAAGGGCTGGCTGTGCC	*Psmb9*	2nd	CO, NCO	B10.A, R209, R5, RJ3, R115
2092U16b10	GTTGTGCAAGATGCCT	*Psmb9*	2nd	NCO	B10
2092U16R209	GTTGTGCAAGATGCCG	*Psmb9*	2nd	NCO	R209, R5, RJ3, R115
2128U18A	ACAGCTTCAAGGGCTCTC	*Psmb9*	2nd	NCO	B10.A
2128U18S	ACAGCTTCAAGGGCTCTG	*Psmb9*	2nd	NCO	B10, RB2, RJ2
BF1	CGAGCACAGACTCTTGTTC	*Hlx1*	1st	CO	B10
CR1	GACATCATTTCCTCACTGGT	*Hlx1*	1st	CO	CAST
BF2	GGCCTGTTTCAGGCTCCG	*Hlx1*	2nd	CO	B10
CR2	CATCATAGCGTCCAGTAATACC	*Hlx1*	2nd	CO	CAST
