# Supplementary material for: Genome-Wide Control of the Distribution of Meiotic Recombination
Source: PLoS Biol. 2009 Feb 17;7(2):e1000035. doi: 10.1371/journal.pbio.1000035 (PMC2642883; doi:10.1371/journal.pbio.1000035)
Supplement: Supplementary file 6 [file pbio.1000035.st004.pdf]

Table S4

MLH1	chromosome 17			chromosome 15		chromosome 18	
**Intervals on SC (%)**	**B10xB10A**	**RB2xB10A**	**SGRxSGR**	**B10xB10A**	**RB2xB10A**	**B10xB10A**	**RB2xB10A**
1-5	0	0	1	0	0	0	0
5-10	1	0	0	2	3	0	0
10-15	3	7	2	2	5	1	1
15-20	3	1	2	8	2	1	1
20-25	3	1	2	10	12	2	6
25-30	3	3	2	22	14	7	7
30-35	7	4	2	9	15	3	7
35-40	4	6	4	6	5	3	6
40-45	4	10	4	15	8	6	7
45-50	6	6	5	21	8	4	13
50-55	4	19	7	15	19	7	14
55-60	9	27	11	23	19	12	28
60-65	18	25	22	16	14	15	24
65-70	12	16	21	20	18	19	17
70-75	21	12	14	13	15	26	21
75-80	30	24	15	21	11	18	12
80-85	38	25	19	29	26	32	32
85-90	27	39	22	36	35	29	12
90-95	31	50	45	28	58	42	40
95-100	21	45	26	29	49	4	17
**Total focus number**	**245**	**320**	**226**	**325**	**336**	**231**	**265**
