# Supplementary material for: Genome-Wide Control of the Distribution of Meiotic Recombination
Source: PLoS Biol. 2009 Feb 17;7(2):e1000035. doi: 10.1371/journal.pbio.1000035 (PMC2642883; doi:10.1371/journal.pbio.1000035)
Supplement: Supplementary file 7 [file pbio.1000035.st005.pdf]

**Table S5**

| MLH1 | chromosome 2 | |
|---|---|---|
| Intervals on SC (%) | B10xB10A | RB2xB10A |
| 0.00 - 2.86 | 0 | 0 |
| 2.86 - 5.71 | 0 | 1 |
| 5.71 - 8.57 | 5 | 3 |
| 18.57 - 11.43 | 8 | 8 |
| 11.43 - 14.28 | 4 | 5 |
| 14.28 - 17.14 | 11 | 10 |
| 17.14 - 20.00 | 14 | 9 |
| 20.00 - 22.86 | 5 | 6 |
| 22.86 - 25.71 | 10 | 8 |
| 25.71 - 28.57 | 12 | 13 |
| 28.57 - 31.43 | 9 | 13 |
| 31.43 - 34.28 | 11 | 11 |
| 34.28 - 37.14 | 14 | 9 |
| 37.14 - 40.00 | 10 | 4 |
| 40.00 - 42.86 | 3 | 9 |
| 42.86 - 45.71 | 3 | 6 |
| 45.71 - 48.57 | 4 | 3 |
| 48.57 - 51.43 | 3 | 3 |
| 51.43 - 54.28 | 5 | 4 |
| 54.28 - 57.14 | 4 | 3 |
| 57.14 - 60.00 | 3 | 2 |
| 60.00 - 62.86 | 5 | 7 |
| 62.86 - 65.71 | 4 | 6 |
| 65.71 - 68.57 | 7 | 0 |
| 68.57 - 71.43 | 3 | 5 |
| 71.43 - 74.28 | 4 | 1 |
| 74.28 - 77.14 | 7 | 3 |
| 77.14 - 80.00 | 3 | 9 |
| 80.00 - 82.86 | 2 | 6 |
| 82.86 - 85.71 | 8 | 6 |
| 85.71 - 88.57 | 10 | 4 |
| 88.57 - 91.43 | 11 | 17 |
| 91.43 - 94.28 | 18 | 18 |
| 94.28 - 97.14 | 26 | 35 |
| 97.14 - 100.00 | 23 | 16 |
| **Total focus number** | **269** | **263** |
