# Supplementary material for: Genome-Wide Control of the Distribution of Meiotic Recombination
Source: PLoS Biol. 2009 Feb 17;7(2):e1000035. doi: 10.1371/journal.pbio.1000035 (PMC2642883; doi:10.1371/journal.pbio.1000035)
Supplement: Supplementary file 8 [file pbio.1000035.st006.pdf]

Table S6

γH2Ax	chromosome 17	
**Intervals on SC (%)**	**B10xB10A**	**RB2xB10A**
1-5	2	2
5-10	13	23
10-15	51	41
15-20	28	46
20-25	33	36
25-30	22	27
30-35	27	18
35-40	33	17
40-45	19	29
45-50	23	16
50-55	13	29
55-60	28	19
60-65	20	28
65-70	30	26
70-75	30	16
75-80	29	15
80-85	35	21
85-90	39	27
90-95	38	30
95-100	36	47
**Total focus number**	**549**	**513**
**Total nucleus number**	**199**	**192**
