# Supplementary material for: Genome-Wide Control of the Distribution of Meiotic Recombination
Source: PLoS Biol. 2009 Feb 17;7(2):e1000035. doi: 10.1371/journal.pbio.1000035 (PMC2642883; doi:10.1371/journal.pbio.1000035)
Supplement: Supplementary file 9 [file pbio.1000035.st007.pdf]

Table S7

Intra Genotype comparaisons	P values	
MLH1 foci, chromosome 17	Chi square	Kolmogorov-Smirnov
B10xB10A mouse n°1 (n:152) vs mouse n°2 (n:93)	0.26	0.80
RB2xB10A mouse n°1 (n:97) vs mouse n°2 (n:80)	0.47	0.10
RB2xB10A mouse n°1 (n:97) vs mouse n°3 (n:45)	0.57	0.29
RB2xB10A mouse n°1 (n:97) vs mouse n°4 (n:98)	0.15	0.58
RB2xB10A mouse n°2 (n:80) vs mouse n°3 (n:45)	0.78	0.98
RB2xB10A mouse n°2 (n:80) vs mouse n°4 (n:98)	0.64	0.44
RB2xB10A mouse n°3 (n:45) vs mouse n°4 (n:98)	0.65	0.60
SGRxSGR mouse n°1 (n:122) vs mouse n°2 (n:104)	0.98	1.00
γH2AX foci, chromosome 17		
B10xB10A mouse n°1 (n:433) vs mouse n°2 (n:116)	0.96	0.62
RB2xB10A mouse n°1 (n:444) vs mouse n°2 (n:69)	0.66	0.49
MLH1 foci, chromosome 15		
B10xB10A mouse n°1 (n:244) vs mouse n°2 (n:81)	0.98	0.67
RB2xB10A mouse n°1 (n:212) vs mouse n°2 (n:124)	0.80	0.27
MLH1 foci, chromosome 18		
B10xB10A mouse n°1 (n:152) vs mouse n°2 (n:79)	0.48	0.21
RB2xB10A mouse n°1 (n:157) vs mouse n°2 (n:108)	0.86	0.25
